# Idiopathic, Pernicious, or Addison's Anæmia

**Published:** 1909-10-23

**Authors:** C. O. Hawthorne

**Affiliations:** Examiner in Medicine and Clinical Medicine in the University of Glasgow, Physician to the North-West London Hospital and to the Central London Ophthalmic Hospital


### HOSPITAL CLINICS.

### IDIOPATHIC, PERNICIOUS, OR ADDISON'S ANÆMIA.

### I.—A SUMMARY AND REVIEW.

By C. O. HAWTHORNE, M.D., M.R.C.P., Examiner in Medicine and Clinical Medicine in the University of Glasgow, Physician to the North-West London Hospital and to the Central London Ophthalmic Hospital.

Questions not a few are raised by the term pernicious anæmia. It has even been debated whether there exists any distinctive, individual disease having an exclusive right and title to the name. The opposing suggestion is that almost any form of anæmia may take on a "pernicious" habit—that, in other words, a pernicious anæmia is merely an anæmia, from whatever cause arising, attended by extreme depreciation of the quality of the blood and tending towards a fatal issue. Others, again, apply the term with even a wider sweep, and include all anæmias in which large nucleated red cells (megaloblasts) appear in the blood; such cells, it is urged, are the expression of retrograde processes in the bone-marrow and afford "proof" of a "pernicious" tendency and direction. But even those who affirm that many anæmias taking origin in recognised causes may become "pernicious" (secondary pernicious anæmia), admit the existence of cases in which a pernicious anæmia exists without any cause that can be discovered, and these cases they distinguish by the term "primary" pernicious anæmia. On the other hand, Dr. Wm. Hunter, whose work on this subject is well known, holds the view that there is one special and distinctive anæmia as opposed to all other anæmias, and this he identifies with the condition described by Addison in 1855 under the name "idiopathic" anæmia. In order to avoid confusion, Dr. Hunter proposes summarily to reject the term pernicious anæmia, and to distinguish the disease to which he formerly applied this name by a new style and title, namely, Addisonian anæmia.

In Addison's original description the anæmia which attracted his attention is said to occur "without any discoverable cause whatever," and to exist apart from previous loss of blood, exhausting diarrhœa, purpura, "renal, splenic, miasmatic, glandular, strumous, or malignant disease." In one or two of its minor features, and as a result of accumulated experiences, the picture thus drawn must certainly be modified. Thus, in a certain number of cases having a general correspondence to Addison's description there is more or less enlargement of the spleen, and sometimes, also, enlargement and tenderness of the liver; hæmorrhages in the skin are at least occasional events, while retinal hæmorrhages are common, and bleeding from mucous membranes is not infrequent; while vomiting, diarrhœa, and other evidences of gastro-intestinal disturbance are now universally recognised among the clinical evidences of the disease. Hence, in the light of wider knowledge, the exclusion of splenic disease, of purpura, and of diarrhœa from the symptomatology of "idiopathic" anæmia cannot be fully maintained. More important than these details there remains the question of a "discoverable cause." And it is here that Hunter's patient and ingenious studies appear to bear fruit, as upon them he bases not only an announcement of the cause of the disease, but also a method for its rational treatment. Briefly, Hunter's position is that in pernicious (Addison's) anæmia there is an abnormal degree of blood destruction (hæmolysis) in the portal circulation—this destruction being due to a specific hæmolytic poison absorbed from the alimentary canal and probably of drain origin. Mere septic conditions of the mouth or other portions of the alimentary canal may cause anæmia (septic anæmia), and even extreme, and perhaps fatal anæmia; but they will not cause Addison's anæmia. Their presence, however, favours the opportunity of the specific poison upon which this latter disease depends, but it is only when the specific infection occurs that the distinctive features of Addison's anæmia appear. The infection may take place at any point of the alimentary canal, and is often early recognised in the tongue, where it produces a glossitis attended by considerable soreness and tenderness, while invasion of the gastric and intestinal areas may be signalled by vomiting, diarrhœa, and abdominal pain. Following the infection comes severe anæmia with clinical evidences of blood destruction, namely lemon tint of the skin, urobilinuria with or without a high colour of the urine, attacks of "biliousness" and occasionally jaundice. The clinical signs of the specific infection, however, are not limited to the site of invasion, namely, the tongue or other portion of the alimentary tract, nor are they restricted to destructive and other changes in the blood. On the contrary, widespread evidences of a general toxæmia may be observed in such symptoms as headache, fever, mental* and bodily prostration, and pain or other sensory disturbances in the trunk or limbs, the degree of these being by no means strictly proportionate to the severity or otherwise of the anæmia; in some instances there are signs of peripheral neuritis, while in others ataxia, loss of knee-jerks, etc., appear as the result of degenerative changes in the spinal cord.† Reviewing all these facts, Hunter claims for Addison's anæmia a position among the specific infective diseases, and he rests the diagnosis neither on the degree of the anæmia (though this is not unimportant), nor on any

* Mental disturbances and even definite delusions, as of suspicion or exaltation, have been recorded in a number of cases of pernicious anæmia (see *Lancet*, June 25, 1904, p. 1808). Doubtless such disturbances are of toxic origin; they appear to be independent of the degree of the anæmia.

† It should be noted that some "nervous" symptoms or complications as hemiplegia, aphasia, coma, and paraplegia may be due to hæmorrhages into one or other part of the brain or spinal cord.

distinctive changes in the blood corpuscles, but on the whole of the phenomena of the disease—glossitic, gastro-intestinal, hæmolytic, febrile, and nervous, all of which he regards, not as consequences of the anæmia, but as the direct results of the invasion of the body by a specific poison.

Further proof that Addison's anæmia is anæmia of an individual and distinctive kind is found in the course pursued by the disease. Promptly after infection, as shown by the development of lesions in the tongue or other parts of the alimentary canal, are to be seen the clinical signs, already enumerated, of hæmolysis and of severe anæmia; and in the most serious cases the condition progresses without interruption to a fatal ending. But in the majority of cases, after some weeks or months, there appear, sometimes with almost critical suddenness, signs of recovery. The patient feels decidedly better, the tongue lesions become quiescent, and the blood value rises, perhaps in the course of a few weeks, to, or near, the normal standard. This favourable change, however, does not endure. After some six to nine months there is a return of the symptoms; and this relapse in most cases proves fatal. In a minority, however, there is more or less complete recovery, which may continue over several months, to be followed, however, by a second and usually fatal relapse. In an occasional instance a patient may survive even a second relapse, and there is reason to anticipate that by destroying septic states of the alimentary canal when these exist, and by the direction of suitable measures against the specific poison responsible for the disease, Addison's anæmia may be brought within the range of successful therapeutics and cease to be either "progressive" or "pernicious."

The clinical picture, as above presented, has, of course, its corresponding post-mortem findings. Of these two are of chief importance. The first is the presence of iron in considerable excess in the liver and to a less extent in the spleen and kidneys. The second pathological fact is an overgrowth of the red bone-marrow, which, in the long bones, may entirely replace the yellow marrow; further, the red marrow is found to contain large numbers of nucleated red cells, some (normoblasts) of the size of normal blood corpuscles, others (megaloblasts) definitely larger than these. Hunter's interpretation of the presence of iron in such large excess in the liver is that the excess is an expression of blood destruction (hæmolysis) in the portal circulation, while the changes in the bone-marrow he regards as compensatory in character.

There are, however, other fashions in which these facts may be interpreted. Thus it has been argued that the excess of iron in the liver may be due to the administration of large quantities of iron in the treatment of the disease, or that it is the result of extravasation of blood* into the liver substance; and some have even contested the accuracy of the observation. Again, in reference to the bone-marrow, it is claimed that the changes here to be seen are not regenerative but retrograde; that so far from being compensatory in character, they indicate, in point of fact, hurry, defect, and inefficiency in the blood-forming process—a reversion, in short, to an early embryonal type of blood-formation. Especial stress in this connection is placed on the presence in large numbers of megaloblasts, which, it is urged, are cells of embryonic type and of but little functional value in the adult. Further, the changes in the bone-marrow on this interpretation are held to be primary —primary, that is, so far as the "perniciousness" of the anæmia is concerned—and thus "pernicious" anæmia is presented as a disease of the bone-marrow; a disease, that is, dependent on a defect of blood-formation, and not, as in Hunter's teaching, due to abnormal blood-destruction. From this difference in interpretation of the pathological facts there follow corresponding differences in nomenclature and diagnosis. For, according to the view just described, the discovery of megaloblasts in the blood in a case of anæmia is a demonstration that the anæmia, however produced, has acquired, as a result of perverted activities in the bone-marrow, a "pernicious" quality; it is of the megaloblastic order, and is, therefore, pernicious, as contrasted with non-pernicious or normoblastic anæmias, in which, although nucleated red blood corpuscles may well be present, they (normoblasts) do not exceed in size the normal red cells. Should there be nothing in the history of the case to explain the megaloblastic degeneration of the bone-marrow and the blood, the diagnosis must be "primary pernicious anæmia"; if, on the other hand, any one of the ordinary causes of anæmia has manifestly been in operation, the resulting anæmia, it must be presumed, has prejudiced the hæmogenetic function of the bone-marrow, and the case must be classified as one of "secondary pernicious anæmia."

Here, then, are two sharply-opposed views. According to one description, there exists a special and distinctive variety of anæmia due to a specific infection and distinguished by evidences of excessive blood destruction, by glossitis, and by gastro-intestinal, febrile, nervous, and other toxic symptoms, these conditions being accompanied by changes of a compensatory order in the bone-marrow. This is the disease presented by Hunter as Addison's anæmia, and in the diagnosis reliance must be placed not merely on the examination of the blood, which may or may not discover megaloblasts, but on the whole of the clinical facts and on the course and movement of the case. As contrasted with Hunter's teaching, the story, on the other hand, is one of more or less extreme anæmia arising from many different causes and sometimes without any cause that can be discovered; various symptoms secondary to the anæmia are present, but the distinctive mark of the condition is the existence in the blood of large nucleated red cells (megaloblasts) which are the outcome of a perverted activity in the blood-forming functions of the bone-marrow.

It cannot be said that either of the above views has

* The tendency to extravasations of blood in severe anæmia is especially insisted on by Professor Stockman, who also contests the specific nature and hæmolytic explanation of pernicious anæmia. He maintains, on the contrary, that any prolonged anæmia, by producing fatty degeneration of the blood-vessels, may cause multiple tissue hæmorrhages, which, in turn, aggravate the anæmia. Hence a vicious circle is established, and the anæmia becomes "progressive" and "pernicious."

received unqualified acceptance, at least in this country. Certainly few, if any, British physicians of experience would venture to urge a diagnosis having such serious prognostic consequences as pernicious anæmia on the single fact that the blood contained more or less numerous megaloblasts; nor would the absence of such cells be held sufficient in itself to exclude pernicious anæmia from the diagnosis.*

In short, the classification of anæmias in two groups—namely, normoblastic or simple on the one side, megaloblastic or pernicious on the other—is recognised as not in harmony with clinical experience. At the same time, it must be admitted that the positive side of Hunter's teaching has by no means received universal assent. Not a few authorities question the doctrine that hæmolysis is the essential and commanding blood-change in pernicious anæmia; and the conditions of the tongue, upon which Hunter places so much stress, appear to have received remarkably little recognition—a record, however, open obviously to various interpretations.

One almost necessary outcome of the conflict of statement and opinion above described is that the diagnosis of pernicious anæmia is apt at the present date to be applied in a somewhat loose fashion, while the diagnostic tests adopted by different physicians are not identical. For so unfortunate a state of matters it is not easy to find a remedy. Hunter, as already indicated, proposes in future to reject the term " pernicious " as applied to any condition of anæmia, and to distinguish the disease having the hæmolytic and infective characters which he describes as " Addison's anæmia." Other severe (sometimes fatal) anæmias he regards as of septic origin (oral, gastric, intestinal). These " septic " anæmias may occur apart from any other disease, or they may complicate various pathological conditions, such, for example, as malignant disease of the stomach and Bright's disease. In their severity they resemble, more or less closely, Addison's anæmia, and as a matter of fact are often mistaken for this disease. The anæmia may be extreme, signs of gastro-intestinal irritation may be present, and hæmorrhages, as well as febrile and nervous symptoms, may complicate the course of the illness. But, in respect both to ætiology and pathology, these " septic " anæmias are separate and apart from Addison's anæmia. They are caused by septic absorption, not by a specific infection; they are not characterised by any abnormal blood-destruction (hæmolysis); and the bone-marrow does not show the changes familiar in Addison's anæmia. Clinically, therefore, " septic " anæmias do not exhibit signs of hæmolysis (lemon tint of skin, urobilinuria, etc.), the course of the disease is not marked by periodic remissions and aggravations of the symptoms, and although the anæmia may be extreme, the outlook is favourable if only the cause of the condition be recognised and removed. Further, there is no glossitis, the examination of the blood finds a low, not, as in Addison's anæmia, a high colour index, and this, provided it is confirmed by repeated examination, may be taken to establish the differential diagnosis. In the examination of the blood the red corpuscles may show poikilocytosis, and normoblasts, and sometimes even megaloblasts, may be present, while the leucocytes vary both in number and character with the circumstances which attend the anæmia. In spite of these distinctions it will be noted that there is a general clinical resemblance between Addison's anæmia and septic anæmia, and confusion is the more likely, as, according to Hunter, septic conditions of the alimentary canal predispose to, and often precede or accompany, the occurrence of the specific infection which is responsible for Addison's anæmia. The latter finds its distinctive characters in clinical and post-mortem evidences of hæmolysis, in hyperplastic changes in the bone-marrow, and in a specific glossitis which, together with the other symptoms, exhibits periodic remissions and aggravations, while examinations of the blood, repeated as may be necessary, show a high hæmoglobin ratio or colour index, great variety in the size of the red corpuscles (microcytes and macrocytes), many distorted corpuscles (poikilocytosis), and usually, but not invariably, both normoblasts and megaloblasts; the white corpuscles with all this being below, or at least not above, the normal numerical standard. Such a clinical picture developing without manifest cause and existing apart from evidences of visceral disease may reasonably be regarded as primary, essential, or idiopathic anæmia, and in all the circumstances Addison's anæmia, as a clinical name for it, may find justification both in history and in convenience. Hunter's suggestion in this direction is not, however, free from difficulties. The term " pernicious " anæmia is well established and will be difficult to uproot, and the new phrase may carry confusions of its own, seeing that Addison's name is already attached to one well-defined clinical picture. Addison's disease and Addison's or Addisonian anæmia are terms so perilously close together in form and sound that they may readily be taken as meaning one and the same thing. Moreover, the conflict will not be composed by a mere adjustment of terms, for, as is here set forth, it deals with facts and with the interpretation of facts, not merely with words and phrases.

While questions of nomenclature are not unimportant, the practitioner of medicine is mainly concerned with more practical issues, and therefore the bearing of the above discussion on clinical work may now be briefly considered. And first it is universally admitted that, whether having the specific nature for which Hunter contends or not, there is a form of anæmia which exists entirely apart from organic visceral disease, is of obscure causation and is associated with an extremely anxious prognosis. In the diagnosis of this disease, though the condition of the blood, and especially the presence of a high colour index, is of importance, many other symptoms occupy a prominent place; and not very infrequently one or other of these symptoms may be the most conspicuous fact in the clinical

* Not every case of serious or even of fatal anæmia existing apart from organic visceral disease shows megaloblasts in the blood, and, on the other hand, these cells may be present in the anæmia of lead-poisoning, in tape-worm anæmia, and occasionally even in chlorosis.

picture. Neither the anæmia, nor some symptom directly dependent on the anæmia, may stand in the forefront; this place may be occupied by vomiting, diarrhœa, abdominal pain, fever, œdema, hæmorrhages (as gastric, uterine, or retinal), sensory nervous disturbances, or organic nervous disease, any one of which on investigation may prove to be an expression of the disease variously known as idiopathic, pernicious, or Addison's anæmia. Secondly, it must be remembered that severe anæmia owning a cause more or less readily overlooked may exist apart from the disease just mentioned. It may, for example, be due to unrecognised or unacknowledged hæmorrhages; bleeding from a duodenal ulcer, where the tarry stools, even if observed by the patient, may fail to carry to his mind a suspicion of blood, is an apt example, and loss of blood from hæmorrhoids occasionally comes into the same category. Rheumatism (particularly in children), tubercle, lead-poisoning, and syphilis are other agencies sometimes responsible for anæmias having no very obtrusive cause, and, exceptionally, intestinal parasites (ankylostomum duodenale, tape worms) hold a similar position. Still further, anæmia of an extreme degree may depend on renal disease or on a malignant growth (especially one affecting the stomach), and if the tumour is not within reach of physical examination an explanation of the anæmia may here well be difficult. And, according to Hunter, some of the most severe anæmias are due to oral, gastric, or intestinal sepsis. In all of these, though there may be a superficial or suggestive likeness to pernicious or Addison's anæmia, a thorough investigation of the facts, including repeated examinations of the blood, will reveal certain points of difference. Hence, while, as already pointed out, symptoms other than the anæmia may be the most conspicuous clinical evidences of this disease, extreme anæmia, even when an explanation is not ready to hand, is not necessarily nor always of the idiopathic, pernicious, or Addisonian order. It is by a recognition of the truth of these two propositions, and by the practice of the clinical conduct which they suggest, that the differential diagnosis of the anæmias can be brought to a successful issue.

